# Determination of 8-OHdG and IL-6 Levels, and of APE1 and XRCC1 DNA Repair Gene Variants, in Patients with Migraine

**DOI:** 10.3390/medicina62061099

**Published:** 2026-06-05

**Authors:** Tuba Gul, Sukran Kaygisiz, Gonca Gulbay, Yasemin Kaya

**Affiliations:** 1 Department of Neurology, School of Medicine, Ordu University, Ordu 52200, Türkiye; tglyzc@hotmail.com (T.G.); skrnkygsz52@gmail.com (S.K.); 2 Department of Medical Biology, School of Medicine, Ordu University, Ordu 52200, Türkiye; 3 Department of Internal Medicine, School of Medicine, Ordu University, Ordu 52200, Türkiye; yaseminkaya@odu.edu.tr

**Keywords:** *APE1*, DNA repair, Migraine, Polymorphism, *XRCC1*

## Abstract

*Background and Objectives:* Migraine is a chronic, throbbing type of headache that affects large populations worldwide. This condition is associated with neuroinflammation. *Materials and Methods*: In this study, polymorphism analyses were performed by KASP PCR. Serum interleukin-6 (IL-6) and 8-hydroxy-2′-deoxyguanosine (8-OHdG) levels were measured using kits based on the enzyme-linked immunosorbent assay (ELISA) principle. *Results:* In the *APE1* Asp148Glu (rs1130409) gene polymorphism analysis, the frequency of the mutant G (Glu) allele was 93.1% and 48.0% in the control and migraine populations, respectively, while the frequency of the wild-type T (Asp) allele was 6.9% and 52.0% (*p* < 0.001). The frequency of the T/T (Asp/Asp) genotype was high in the migraine group (*p* < 0.001), while the frequency of the G/G (Glu/Glu) genotype was higher in the control group at 86.2%, compared to the migraine group (*p* < 0.001). The total frequency of the T/G+ G/G (Asp/Glu+Glu/Glu) composite genotype was determined to be 65.9% in the control group and 34.1% in the migraine group (*p* < 0.001). There was no statistical difference in allele and genotype frequency between the control and migraine groups for the *XRCC1* Arg399Gln (rs25487) gene polymorphism. Serum 8-OHdG and IL-6 levels were comparable between the groups, with no statistically significant differences observed. *Conclusions:* Future studies with larger and more homogeneous populations are needed to further elucidate the potential interactions between inflammatory processes and DNA damage in migraine. Consideration of attack duration and environmental exposures may improve interpretation of biomarker variability. Expanding the analysis to additional DNA repair gene polymorphisms may also contribute to a better understanding of the molecular background of migraine and the evaluation of potential biomarkers.

## 1. Introduction

Migraine is a pervasive neurovascular pathology affecting approximately 15% of the global population and is recognized as a formidable public health challenge due to its debilitating nature [1,2,3,4]. Clinically, it is characterized by paroxysmal episodes of moderate-to-severe, typically hemicranial and pulsating headaches, frequently accompanied by sensory hypersensitivities such as photophobia, phonophobia, and autonomic disturbances [5,6]. Epidemiological assessments consistently categorize migraine as a primary driver of neurological disability, particularly among women of reproductive age [1,2,7]. Although its precise etiology remains under investigation, current paradigms suggest that the disorder stems from the pathological activation of the trigeminovascular system and dysfunctional neuronal signaling within the central nervous system [2,6,8].

The role of oxidative stress in many diseases, particularly obesity, diabetes, cancer, and viral infections [1,9,10,11,12], is quite clear. A growing body of evidence suggests that oxidative stress plays an increasingly significant role as a key factor in the pathophysiological progression of migraine and its potential chronicity [13,14,15,16].

Oxidative stress results from an imbalance between the production of reactive oxygen species (ROS) and the capacity of endogenous antioxidant defense mechanisms. Excessive ROS generation can damage key cellular macromolecules, including DNA, lipids, and proteins, thereby facilitating neuronal sensitization and promoting neurogenic inflammatory responses [1,13,14]. In line with this mechanism, several studies have reported elevated circulating levels of pro-inflammatory cytokines such as interleukin-6 (IL-6), interleukin-8 (IL-8), and tumor necrosis factor-α (TNF-α) in individuals with migraine, suggesting the presence of a sustained inflammatory milieu within the nervous system [17,18]. Conversely, activation of the nuclear factor erythroid 2–related factor 2 (Nrf2) signaling pathway, as well as interventions targeting anti-inflammatory and antioxidant mechanisms such as magnesium supplementation, may mitigate oxidative damage and suppress pro-inflammatory cytokine expression, thereby exerting potential neuroprotective effects [2,13].

DNA damage is repaired by various mechanisms. Base excision repair (BER) and nucleotide excision repair (NER) are among these mechanisms. BER is the primary mechanism for repairing small lesions caused by base modifications, methylation agents, and small insertions, and for removing oxidized DNA bases [19,20]. The NER pathway is responsible for repairing lesions such as pyrimidine dimers and other large chemical additions, primarily UV light damage [21,22,23]. X-ray repair cross-complementing group 1 (*XRCC1*) and apurinic/apyrimidinic endonuclease (*APE1*) are key genes in the BER pathway [21].

Genetic susceptibility also represents a critical component in the development of migraine, with heritability estimates ranging from approximately 30% to 60% [6,24]. Emerging research has expanded the focus beyond ion channelopathies to include defects in DNA maintenance pathways, particularly the BER system, as potential contributors to migraine susceptibility. Polymorphisms in genes such as *APE1* and *XRCC1*, which are vital for repairing oxidative DNA lesions, may compromise neuronal genomic integrity and exacerbate cellular vulnerability [24,25].

This study aimed to demonstrate the effect of inflammation and oxidative stress in migraine cases and to investigate the *APE1* (Asp148Glu) and *XRCC1* (Arg399Gln) DNA repair gene polymorphisms.

## 2. Material and Methods

### 2.1. Study Groups

In this study, blood samples were collected from patients who presented with migraine, were under follow-up at the neurology outpatient clinic, and signed the consent form at the Neurology Department of the Education and Research Hospital affiliated with Ordu University Faculty of Medicine between 20 September 2025 and 20 November 2025.

The study included patients aged 18–55 years who had been diagnosed with migraine according to the ICHD-3 diagnostic criteria, as well as healthy controls in the same age group. Participants with systemic chronic diseases other than regulated hypertension, a history of autoimmune, infectious, malignant, or hematological diseases, tobacco and/or alcohol use, use of anti-inflammatory or immunomodulatory drugs within the last 3 months, pregnancy or breastfeeding, a history of previous brain surgery or trauma, or a diagnosis of genetic disease were excluded from the study.

A total of 54 participants (29 healthy controls and 25 cases) were included in this study. Blood was taken into 1 tube containing separating gel for IL-6 and 8-OHdG ELISA study and into an EDTA tube for gene variants.

Ethical approval for the study was obtained from the Ordu University Faculty of Medicine Institutional Review Board Ethics Committee (dated: 12 September 2025, decision number: 2025/295). All subjects provided informed consent prior to their inclusion in the study.

### 2.2. Determination of APE1 (Asp148Glu. rs1130409) and XRCC1 (Arg399Gln. rs25487) Gene Variants

#### 2.2.1. DNA Extraction

Genomic DNA was isolated from peripheral blood leukocytes using the QIAsymphony DSP DNA mini kit (Qiagen, Hilden, Germany) according to the manufacturer’s instructions and stored at −20 °C until analysis. Invitrogen Qubit 4 fluorometer (Thermo Fisher Scientific, Waltham, MA, USA) was used to measure the purity and concentration of extracted DNA samples.

#### 2.2.2. The Validation of Plate Reader Functionality and KASP-PCR Reaction

Primers corresponding to SNP positions were designed manually. The relevant gene sequences were retrieved from the NCBI database (https://www.ncbi.nlm.nih.gov). Prior to genotype determination, the KASP genotyping validation kit (LOW ROX KASP TF Validation kit, LGC, Teddington, UK) was used to validate the plate reader device (BioRad CFX96 Real-Time PCR, Bio-Rad Laboratories, Inc., Hercules, CA, USA). In accordance with the manufacturer’s protocol, the compatibility and readability of the plate reader were validated [26]. The kit consists of three tubes containing diluted fluorophores: FAM, HEX, and HEX/FAM. The tubes were briefly vortexed and dispensed into a microtiter plate. The tubes were briefly vortexed and transferred to a microtiter plate. Subsequently, the provided DNA samples were used to run the kit protocol with KASP reagents. For the KASP PCR reaction, a mixed reaction solution containing forward-1, forward-2, and reverse primers, KASP 2x Master mix, and genomic DNA was prepared and transferred to PCR tubes. The tubes were then closed and placed in the device.

All samples were successfully genotyped, yielding a genotyping call rate of 100%. Genotype calls were reviewed for quality control. We also clarified the allele nomenclature throughout the manuscript to avoid confusion between nucleotide substitutions and corresponding amino acid variants.

### 2.3. ELISA

For IL-6 and 8-OHdG, blood samples taken from the control and case groups were centrifuged at 12.000 rpm at 4 °C for 20 min. Serum was separated and stored at −80 °C for further study. It was used to measure 8-OHdG and IL-6 levels.

#### 2.3.1. 8-OHdG Assay

Accordingly, the 8-hydroxy-2′-deoxyguanosine (8-OHdG) protein level in blood samples from migraine patients and the control group was investigated using an enzyme-linked immunosorbent assay (ELISA) kit according to the manufacturer’s protocol (BT LAB, China ELISA, Shanghai, China).

Absorbance values (450 nm) were measured based on the user manuals for the relevant kits. A Biotek Epoch 2 microplate reader (Agilent Technologies, Inc., Santa Clara, CA, USA) was used for this purpose. Results were analyzed using Gen5 software.

#### 2.3.2. IL-6 Assay

Interleukin-6 (IL-6) protein level in blood samples of migraine patients and the control group was investigated by an enzyme-linked immunosorbent assay kit (ELISA) according to the kit protocol (BT LAB, China ELISA, Shanghai, China).

Absorbance measurements at 450 nm were performed in accordance with the protocols provided in the respective kit manuals. The readings were obtained using a Biotek Epoch 2 microplate reader (Agilent Technologies, Inc., Santa Clara, CA, USA), and the resulting data were analyzed with Gen5 software.

### 2.4. Statistical Analysis

Chi-square analysis of the distribution of *APE1* Asp148Glu and *XRCC1* Arg399Gln genotypes in our populations indicated that all of the alleles were in Hardy–Weinberg equilibrium (*p* > 0.05).

The normality of the data distribution was assessed using the Kolmogorov–Smirnov test. Student’s *t*-test was used to compare continuous variables that showed a normal distribution, while the Mann–Whitney U test was used to compare variables that did not show a normal distribution. Categorical variables were analyzed using the chi-square test. In chi-square analysis, Fisher’s exact test was applied instead of Pearson’s chi-square test when the expected cell frequency was less than 5. Numerical variables were expressed as mean ± standard deviation and median (minimum–maximum), while categorical variables were expressed as percentages (%). All analyses were evaluated at a 95% confidence interval, and statistical significance was set at *p* < 0.05.

## 3. Result

### 3.1. Analysis of Laboratory Data

The mean age of the migraine group was 39.92 ± 8.47, while that of the control group was 29.27 ± 9.71 (*p* < 0.001). The migraine group consisted of 36.0% male and 64.0% female, while the control group consisted of 75.9% female and 24.1% male. Fasting blood glucose, TSH, hemoglobin, urea, and creatinine levels in the control group were 91.03 ± 6.94, 1.54 ± 0.54, 13.6 ± 1.46, 11.5 ± 4.77, and 0.73 ± 0.16, respectively, while in the migraine group they were 103.33 ± 27.14, 1.88 ± 0.93, 13.77 ± 1.40, 12.02 ± 4.88, and 0.76 ± 0.14, respectively (Table 1).

### 3.2. Genotyping of APE1 (Asp148 Glu, rs1130409)

After the end of the KASP PCR reaction, allelic discrimination analysis was performed using the BioRad CFX96 Real-Time PCR device software. A KASP-PCR protocol was performed to determine the *APE1* Asp148Glu polymorphism. Heterozygous (TG, Asp/Glu), wild-type (TT, Asp/Asp), and homozygous mutant (GG, Glu/Glu) genotypes were determined (Table 2).

For the *APE1 Asp148Glu* gene polymorphism, the mutant G (Glu) allele frequency was 93.1% and 48.0% in the control and migraine populations, respectively, while the T (Asp) wild-type allele frequency was 6.9% and 52.0% (*p* < 0.001).

The Asp/Asp (T/T), Asp/Glu (T/G), and Glu/Glu (G/G) genotype frequencies were 0%, 13.8%, and 86.2%, respectively, in the control group, while they were 40%, 24.0%, and 36.0%, respectively, in the migraine group. In the migraine group, the Asp/Asp (T/T) genotype frequency was high (*p* < 0.001), while in the control group, the Glu/Glu (G/G) genotype frequency was higher than in the migraine group at 86.2% (*p* < 0.001).

The total frequency of the Asp/Glu+Glu/Glu (T/G+ G/G) composite genotype was determined to be 65.9% in the control group and 34.1% in the migraine group (*p* < 0.001).

### 3.3. Genotyping of XRCC1 (Arg399Gln, rs25487)

After the end of the KASP PCR reaction, allelic discrimination analysis was performed using the BioRad CFX96 Real-Time PCR device software. A KASP-PCR protocol was performed to determine the *XRCC1* Arg399Gln polymorphism. Heterozygous (GA, Arg/Gln), homozygous wild type (GG, Arg/Arg), and homozygous mutant (AA, Gln/Gln) genotypes were determined (Table 3).

For the *XRCC1* Arg399Gln gene polymorphism, the G (Arg) wild-type allele frequency in the control and migraine groups was 6.9% and 2.0%, respectively, while the mutant A (Gln) allele frequency was 93.1% and 98.0%, respectively (*p* = 0.227).

The Arg/Gln (G/A) heterozygous mutant and Gln/Gln (A/A) homozygous mutant genotype frequencies were 13.8% and 86.2% in the control group, respectively, while they were 4.0% and 96.0% in the migraine group. The Arg/Arg (G/G) homozygous wild-type genotype was not observed in either the control or migraine case group.

The total frequency of the Arg/Gln (G/A) + Gln/Gln (A/A) composite genotype was determined to be 100% in both the control and migraine groups.

### 3.4. 8-OHdG Analysis

The serum 8-OHdG levels of the control and case groups are shown in Table 4. Accordingly, the mean 8-OHdG biomarker levels in the control and migraine patient groups were 65.91 ± 30.43 ng/mL and 80.53 ± 50.23 ng/mL, respectively, and there was no statistically significant difference.

### 3.5. IL-6 Analysis

The serum IL-6 levels of the control and case groups are presented in Table 4. The mean IL-6 concentration was 920.356 ± 316.262 ng/mL in the control group and 962.11 ± 307.601 ng/mL in the case group. No statistically significant difference was observed between the two groups in terms of IL-6 levels.

## 4. Discussion

Migraine is a progressively better-recognized disorder in which inflammation and oxidative stress are thought to play a central role in its pathogenesis. The interaction of inflammatory processes with oxidative stress, the disruption of DNA integrity, and the triggering of cellular response mechanisms are considered one of the fundamental biological mechanisms in the development and progression of the disease. However, within the scope of the data we could access, there appear to be no studies evaluating the relationship between migraine and inflammation, oxidative stress, and DNA repair gene polymorphisms (*XRCC1* Arg399Gln, *APE1* Asp148Glu) within the same study and using a comprehensive approach. To the best of our knowledge, there are limited studies in the literature that simultaneously evaluate these three fundamental pathobiological processes implicated in migraine. In this respect, the present study provides additional data that may help to better elucidate the molecular and cellular mechanisms underlying the disease and contribute to the existing body of evidence.

Yigit et al. reported in their study that plasma lymphocyte DNA damage, TOS, MDA levels, and OSI values were significantly higher in migraine patients compared to the control group, while TAS levels were markedly lower in migraine patients compared to the control group [27]. Studies have found pro-inflammatory cytokines such as interleukin-1 (IL-1) and IL-6 at the onset of migraine and throughout the duration of migraine. In addition, IL-6 levels were found to rise in the first hours of an attack, including IL-10, IL-8, and TNF-α levels [1,28]. This study found no significant difference in serum IL-6 and 8-OHdG levels. The fact that serum samples were not collected during the attack period in our study may have resulted in missing the period when the inflammatory response was at its peak. The fact that cytokines associated with the acute phase response, such as IL-6, rise during the attack and return to normal during the remission period may explain why no significant difference was found between the patient and control groups.

Human DNA repair gene profiles are quite heterogeneous and show tolerance to different levels of nucleotide variability/polymorphism [29]. *XRCC1* is an important protein involved in single-strand breaks and the BER pathway and is known to be responsible for repairing DNA damage caused by free radicals, alkylating agents, and ionizing radiation [21].

Karpuzoglu et al. found no difference between patient and control groups in terms of *XRCC1* Arg399Gln polymorphism in Alzheimer’s patients [30]. Aslan et al. found a significantly higher frequency of both the homozygous mutant Gln/Gln genotype and the total mutant genotype frequency in Parkinson’s patients with the *XRCC1* Arg399Gln polymorphism [31]. In this study, no difference was found between the control and migraine groups in terms of the *XRCC1* Arg399Gln gene polymorphism.

It is known that the expression of the *APE1* gene plays a key role in the removal of mispaired bases, is induced under damaging stress conditions such as UV and ROS, and increases endonuclease activity [32]. A study on the *APE1* Asp148Glu polymorphism reported increased sensitivity to ionizing radiation in individuals carrying the mutant allele (G) [33]. In another study examining the *APE1* Asp148Glu polymorphism, no difference was found between Alzheimer’s patients and control groups [30].

In this study, the homozygous mutant genotype for the *APE1* Asp148Glu gene polymorphism, the total mutant genotype (Asp/Glu+Glu/Glu), and the frequency of the mutant allele (G) were higher in the control group than in the migraine group. However, the wild-type genotype (Asp/Asp) was also statistically significantly higher in the migraine group. This observation indicates a difference in allele and genotype frequencies between groups; however, it should be interpreted cautiously and does not necessarily imply a protective effect of the mutant allele or a lack of association between the studied polymorphisms and migraine. Given the limited sample size, these findings may also reflect random variation [34,35]. Therefore, the results should be considered preliminary and require confirmation in larger, well-powered studies.

In this study, the mean age in the migraine group was significantly higher than in the control group. The significant age difference between the groups is a factor that must be carefully considered when interpreting the current findings. Because age can influence inflammatory and oxidative stress pathways, the observed genetic associations may have been partially influenced by this imbalance [36]. Therefore, the results should be interpreted not as definitive evidence of a causal relationship, but as preliminary and exploratory unadjusted observations.

This study has several limitations that should be acknowledged. First, the relatively small sample size limits the statistical power of the analyses and reduces the ability to detect modest genetic effects; therefore, the findings should be interpreted as exploratory and hypothesis-generating rather than confirmatory. Second, there is a difference in the age distribution between the case group and the control group. Since the analysis was not adjusted for age, it is not possible to rule out residual confounding. Third, there has been no comprehensive clinical characterization of migraine patients. Although the diagnosis of migraine was made according to ICHD-3 criteria, the migraine subtype (with or without aura), the distinction between episodic and chronic migraine, attack frequency, disease duration, pain intensity, use of preventive treatment, use of acute medications, the timing of the most recent migraine attack, and the relationship between blood draws and migraine attacks were not systematically analyzed within the scope of this study. This study was conducted at a single center; this may limit the generalizability of the findings. Despite these limitations, the study provides preliminary data that could contribute to the current limited literature and serve as a foundation for future, larger-scale studies.

## 5. Conclusions

This study investigated the role of *APE1 Asp148Glu* and *XRCC1 Arg399Gln* gene polymorphisms in migraine. The distribution of the APE1 mutant allele and genotype frequencies between the study groups is reported descriptively. No significant association was found between the patient and control groups regarding *XRCC1* gene polymorphisms. Furthermore, no significant association was found in terms of IL-6 levels and the 8-OHdG DNA damage marker. The limited findings obtained highlight the small sample size and the genetic and environmental heterogeneity. Our study is a pioneering effort; it is recommended that future studies, utilizing larger, more homogeneous, and age-matched sample groups that take into account pain attack durations and environmental exposures, examine in greater detail the relationships between DNA repair gene polymorphisms associated with different pathways and inflammation and DNA damage. This approach could contribute to a better understanding of the molecular mechanisms of migraine and to the evaluation of potential biomarkers for risk assessment.

## Figures and Tables

**Table 1:** Comparison of age, gender, and biochemical parameters.

	Control n (%)	Migraine n (%)	*p*-Value
Age, year	29.27 ± 9.71	39.92 ± 8.47	<0.001
Gender n(%)			
Male	7 (24.1)	6 (36)	0.384
Female	22 (75.9)	16 (64)	
Fasting blood glucose, mg/dL	91.03 ± 6.94	103.33 ± 27.14	0.119
TSH, mUI/mL	1.54 ± 0.54	1.88 ± 0.93	0.103
Hemoglobin, gr/dL	13.6 ± 1.46	13.77 ± 1.40	0.068
Urea, mg/dL	11.5 ± 4.77	12.02 ± 4.88	0.695
Creatinine, mg/dL	0.73 ± 0.16	0.76 ± 0.14	0.532

TSH: Thyroid-Stimulating Hormone.

**Table 2:** Distribution of genotype and allele frequencies in the control group and patients with migraine (*APE1 Asp148Glu*).

*APE1* *Asp148Glu*	Control n (%)	Migraine n (%)	*p*-Value	X^2^	OR (95% CI)
T/T (Asp/Asp)	0 (0)	10 (40.0)	<0.001	18.009	1.667 (1.210–2.295)
T/G (Asp/Glu)	4 (13.8)	6 (24.0)	0.485	0.927	1.974 (0.487–7.994)
G/G (Glu/Glu)	25 (86.2)	9 (36.0)	<0.001	14.51	0.90 (0.24–0.342)
T/G+ G/G (Asp/Glu+Glu/Glu)	29 (65.9)	15 (34.1)	<0.001	14.23	1.667 (1.210–2.295)
T (Asp) allele frequency	4 (6.9)	26 (52.0)			
G (Glu) allele frequency	54 (93.1)	24 (48.0)	<0.001	27.22	0.068 (0.0215–0.2175)

**Table 3:** Distribution of genotype and allele frequencies in the control group and patients with migraine (*XRCC1 Arg399Gln*).

*XRCC1* *Arg399Gln*	Control n (%)	Migraine n (%)	*p*-Value	X^2^	OR (95% CI)
G/G (Arg/Arg)	0 (0)	0 (0)	-	-	-
G/A (Arg/Gln)	4 (13.8)	1 (4.0)	0.358	1.651	0.260 (0.027–2.500)
A/A (Gln/Gln)	25 (86.2)	24 (96.0)	0.358	1.533	3.840 (0.400–36.864)
G/A+A/A (Arg/Gln+Gln/Gln)	29 (100.0)	25 (100.0)	-	-	-
G (Arg) allele frequency	4 (6.9)	1 (2.0)			
A (Gln) allele frequency	54 (93.1)	49 (98.0)	0.227	1.45	3.629 (0.392–33.593)

**Table 4:** Comparison of groups in terms of 8-OHdG and IL-6.

	Control	Migraine	*p*-Value
8-OHdG	65.91 ± 30.43	80.53 ± 50.23	0.195
IL-6	920.356 ± 316.262	962.11 ± 307.601	0.626

## Data Availability

Data are contained within the article.
